# A bibliometric analysis of food safety governance research from 1999 to 2019

**DOI:** 10.1002/fsn3.2220

**Published:** 2021-03-03

**Authors:** Cong Shen, Mingxia Wei, Yilong Sheng

**Affiliations:** ^1^ School of Management Henan University of Technology Zhengzhou China; ^2^ School of Management Wuhan Institute of Technology Wuhan China

**Keywords:** bibliometric research, food safety governance, income level, research hot spot, research trend

## Abstract

Although the number of food governance‐related studies increased rapidly in the recent decade, the current academic research still lacked systematic integration of food safety governance. To clarify the development trends of research therein, this study summarized research articles concerning food safety governance by the Web of Science Core Collection. An in‐depth bibliometric analysis was then conducted through CiteSpace to summarize the current characters and hot spots of food safety governance research, and predicted future research trends. Results showed that food safety governance was multidisciplinary, which included environmental science, food science, economics, and agriculture. The United States had the largest number of relevant articles, and Wageningen University was the most influential scientific research institution. Among all the journals in this field, *Food Policy* ranked the first in publication volume and co‐citation frequency. The development of food safety governance research was divided into three processes, namely the separate formulation of the standards for public and private sectors, the joint implementation of these standards, and co‐governance by multiple sectors. The most popular research hot spots in this field were food safety policy integration and public–private partnership of food safety governance. Lower‐ and middle‐income countries focused more on food supply and food system design, and regrettably not on food safety. Higher‐income countries cared more about food safety and food nutrition. Besides, researchers of higher‐income countries also concentrated on consumers' voices in participating in food safety governance. Food safety co‐governance, online food governance, the willingness to buy safe food, and food safety governance under pandemics were considered as future research directions.

## Graphical abstract

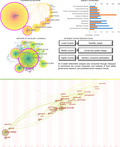

## 1 INTRODUCTION

Food is the paramount necessity of people, and food safety is the top priority. The control of food safety is crucial to the government's image, the survival of enterprises, public interest, and social stability (Kumar et al., 2017). Food safety risks may occur in every link in the food supply chain, including planting, breeding, production, processing, storage, transportation, and sales. After continuous accumulation and transmission upstream of the supply chain, these unsafe factors are likely to induce food safety problems downstream, endanger consumers' lives, and health. At present, foodborne pathogens such as *Colibacillus*, *Salmonella*, and *Norovirus* contaminate agricultural food products (meat, vegetables, and dairy products) worldwide, thereby posing severe challenge to public health (Newell et al., 2010).

The issue of food safety has long been of concern among politicians and scholars around the world. Despite the continuous optimization and improvement of the detection technology, legal system, governance institutions, and other aspects in recent years, global food safety incidents still occur (Downing et al., 2017). Taking China as an example, although the central government took a series of measures against the melamine substandard milk powder incident in 2008, such as the urgent promulgation and implementation of the *PRC Food Safety Law*, actively restructuring and reforming food safety governance institutions, and the introduction of advanced testing technology and equipment, it still failed to curb the long‐term grim situation in the food industry (Kang, 2019; Liu et al., 2019). After that incident, food safety incidents such as waste oil, clenbuterol hydrochloride, poisoned rice, zombie meat, and African swine fever still occurred frequently. It was concluded that food quality and safety could not be guaranteed even with advanced detection technology, a sound legal system, and a perfect supervision regime. To a large extent, the continuous supply of safe food also depended on whether the food safety management process was effective, as well as the standard behaviors of producers and operators. The gradual improvement of the above aspects required an in‐depth study of food safety governance.

In order to provide researchers a clear understanding of current emphasis and future trends on food safety governance research, it is necessary to conduct a systematic analysis of relevant literature in the existing research field. In the past two decades, there were many papers published on food safety governance in various academic journals, covering the perspectives of management dilemma (Glamann et al., 2017; Scott et al., 2018), management models (Garcia Martinez et al., 2007; Zanella et al., 2018; Zhu et al., 2019), and consumer behavior (My et al., 2017; Zhu et al., 2017); however, the lack and inadequacy of the existing literature was not only the relevant exploration of relationships among countries, institutions, journals, and authors in the field of food safety governance, but also the systematic analysis of the research hot spots and trends therein. In this regard, the authors of this research expect to use a bibliometric method to collect food safety governance‐related documents, then analyze the research character, context, and hot spots of these documents, and further predict possible future research trends. Meanwhile, this research was expected to discover the focus of food safety governance in different income level countries to help policymakers formulate food safety‐related policies. To have a more comprehensive understanding of food safety‐related research, food safety was defined as a broader definition in this study, which included food quantity safety, food quality safety, and food nutrition safety.

## 2 DATA SOURCES AND METHODOLOGY

### 2.1 Data collection and processing

The data were retrieved from Science Citation Index Expanded (SCI‐E) and Social Sciences Citation Index (SSCI) of the Web of Science Core Collection. This database constitutes the most comprehensive and frequently used scientific database in most disciplines. The data covered all the literature related to food safety management in these two databases from January 1999 to December 2019. The retrieval method is as follows. As food security, food safety, and food quality (including food nutrition) were considered as key aspects of food systems with important implications for public health (Walls et al., 2019), the key terms were designed as follows. The search terms included TI (Title) = [(“food safety”, OR “food security”, OR “food quality”, OR “agricultural product safety”) AND (“regulation” OR “co‐regulation” OR “governance” OR “supervision” OR “management”)]. The types of article were paper and review, written in English. A total number of 745 articles were searched, and 740 valid documents were finally obtained after eliminating duplicates in CiteSpace V.5.6.R3.

### 2.2 Methods of bibliometric analysis

The bibliometric method was originally used to review the subjects, authors, scientific research institutions, journals, and research area of literature, and to overcome the deficiency of subjectivity in peer review and expert judgment with objective quantitative indicators. Due to the large number of literatures involved, traditional scientometric measurement is not applicable to this study. The information visualization method, to a certain extent, can systematically summarize and research the literature collected from multiple data sources and present them in the form of a knowledge map. Professor Chen Chaomei, the CiteSpace developer, is a professor at the School of Information Science, Drexel University (Chen & Song, 2019). CiteSpace is considered as a useful tool for visual bibliometric analysis, which expands and improves the traditional bibliometric method. Firstly, research hot spots could be easily found out through the cluster analysis (a technique which aims to find internal structure among data (Madani, 2015)). Secondly, the co‐occurrence and co‐citation of different node types such as countries, institutions, authors, keywords, and cited literature could also be clearly displayed (Chen et al., 2010). This enables researchers to grasp the research focus and research trends more accurately. In this study, we use the cosine function of CiteSpace to handle strengths between cluster links and nodes. It is generally acceptable that frequency and centrality are important analytical parameters when discussing such results.

## 3 RESULTS AND DISCUSSION

**FIGURE 1:**
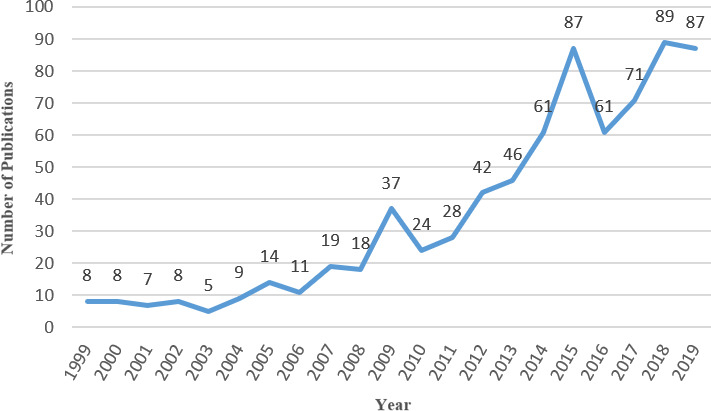
Publication output performance in food safety governance from 1999 to 2019

From the perspective of time distribution, the number of relevant studies published on food safety management showed an overall growth trend from 1999 to 2019. The growth rate was relatively fast after 2008, and there was a surge in the number of papers in 2009 and 2015 (Figure 1). The reason behind this might be related to the serious food safety and public health incidents that occurred before these 2 years. By reviewing the relevant years, it was found that in 2008, serious food safety incidents occurred in China's dairy industry, the United States' meat‐processing industry and fast‐food industry. In 2014, China's meat‐processing industry, the Australian dairy industry, and the European meat‐processing and catering service industry also suffered several serious food safety incidents. Meanwhile, the sudden outbreak of major public health events such as H1N1 in 2009, H7N9 in 2013, and MERS in 2015 also had a certain influence on global food safety (Aiyar & Pingali, 2020; Harder et al., 2016).

### 3.1 Country, institution, and subject category analysis

**FIGURE 2:**
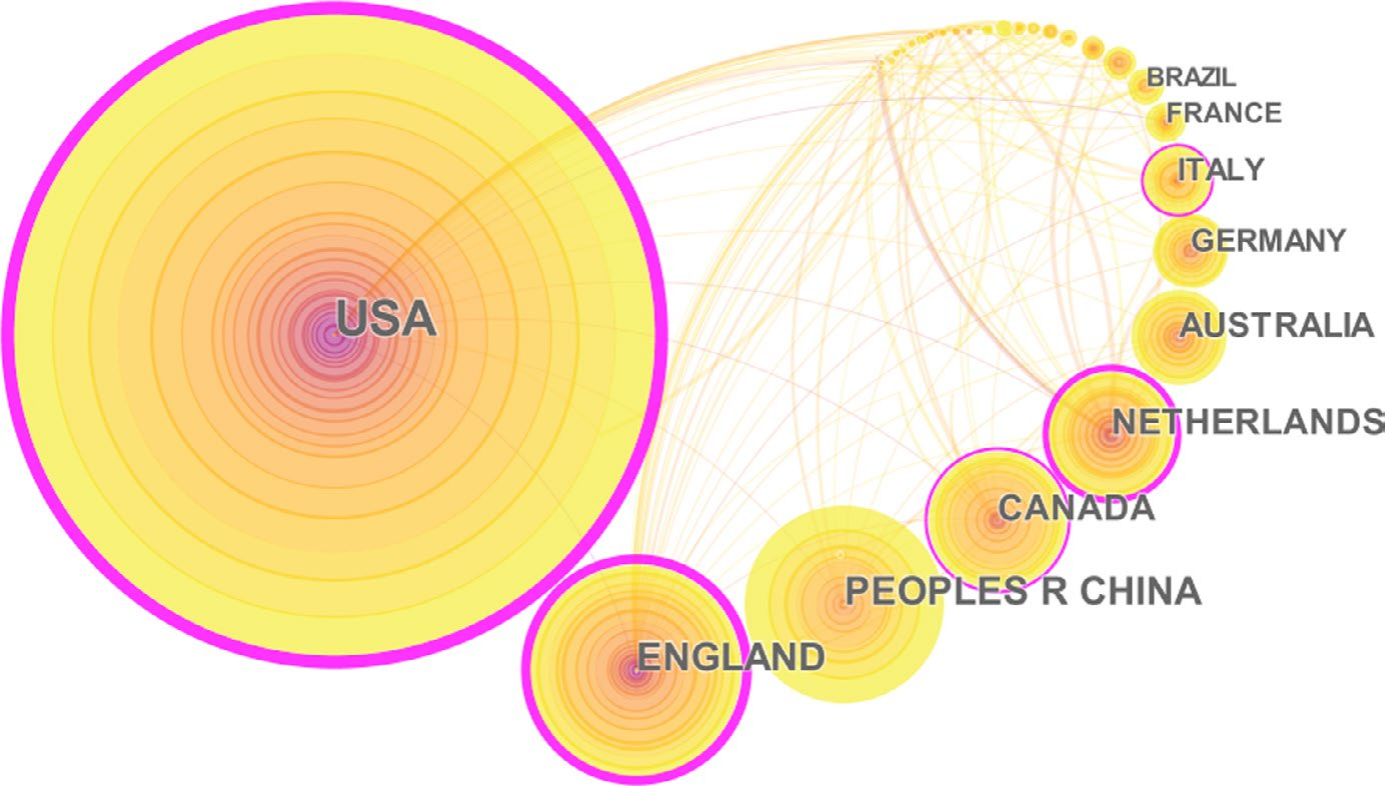
The cooperation network of the productive countries in food safety governance from 1999 to 2019

In Figure 2, the 45 nodes and 132 links reflected the research status in different countries. The size of the circle represented the importance of each country in this research field, and the thickness of the connecting lines indicated the cooperation intensity between countries. It showed that the research on food safety governance was relatively concentrated in European and American countries. Among them, the United States ranked first with 253 articles published, accounting for 34.2%. This was then followed by the UK and China with 89 and 80 articles published, accounting for 12.0% and 10.8% respectively. In addition, Canada and the Netherlands were among the top five countries. The cooperation intensity of the national cooperation network can be derived from the parameter of centrality. It was found that in the ranking of cooperation intensity of countries, the top five countries were the USA (0.67), the UK (0.53), the Netherlands (0.23), Canada (0.17), and Italy (0.12).

**FIGURE 3:**
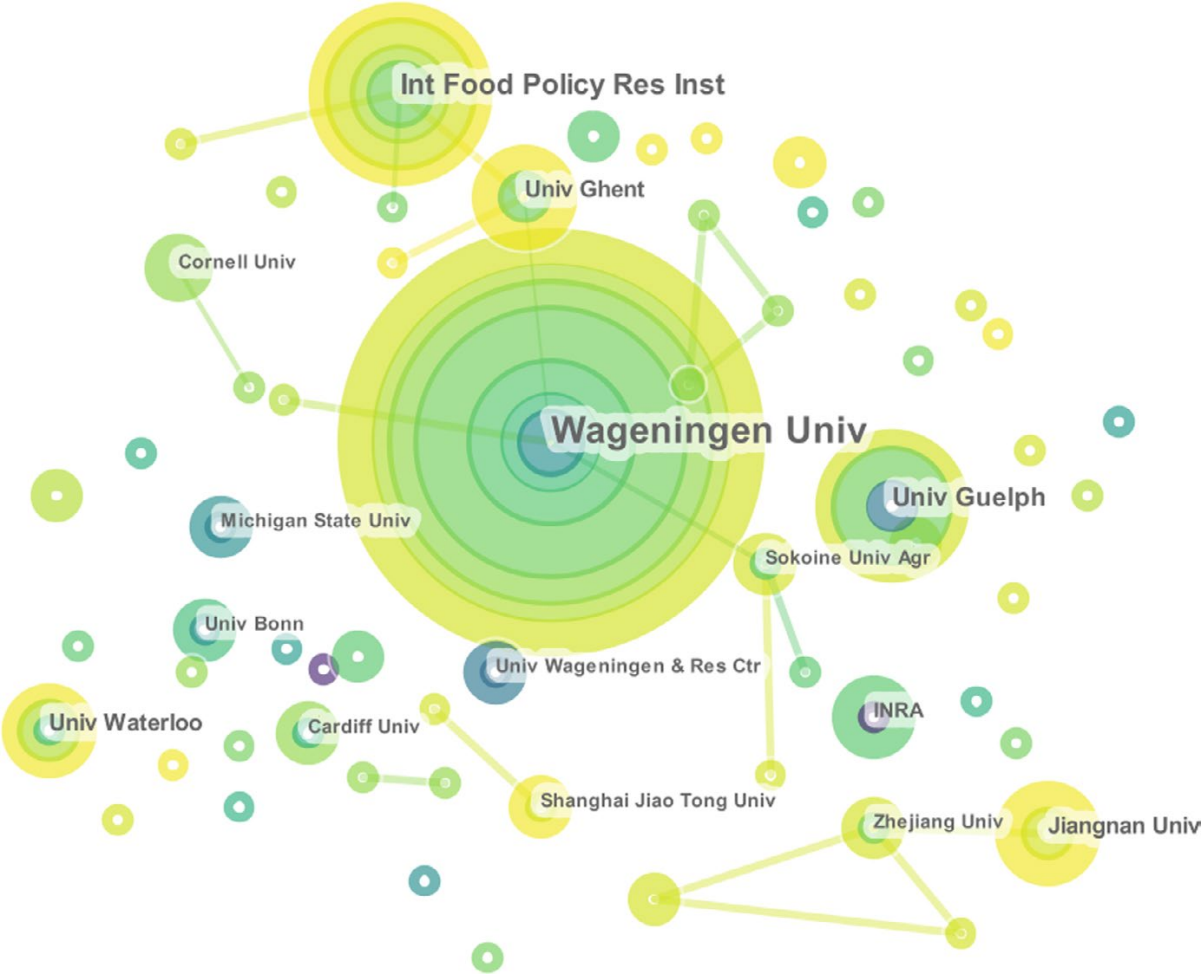
The cooperation network of institutions in food safety governance from 1999 to 2019

Figure 3 illustrates the cooperation network of institutions in this research field. Wageningen University (Candel, 2014) (25 articles), International Food Policy Research Institute (Chen et al., 2015) (11 articles), and University of Guelph (Garcia Martinez et al., 2007) (nine articles) were the top three main productive institutes. This was then followed by University of Waterloo (Clapp & Murphy, 2013), Ghent University (Luning et al., 2015), and Jiangnan University (Wu et al., 2018a), each with six articles. The French National Institute for Agricultural Research (INRA) (Crespi & Marette, 2001) had a total of five published articles. Other important institutions, such as Michigan State University (Dorosh et al., 2009), University of California Davis (Tscharntke et al., 2012), Cornell University (Gregory et al., 2016), Cardiff University (Sonnino, 2016), Shanghai Jiao Tong University (Han & Yan, 2019), Zhejiang University (Zhou et al., 2015), Wageningen University and Research Centre (Valeeva et al., 2007), and University of Bonn and Sokoine University of Agriculture (Alphonce et al., 2014), shared an equal number of four publications.

**FIGURE 4:**
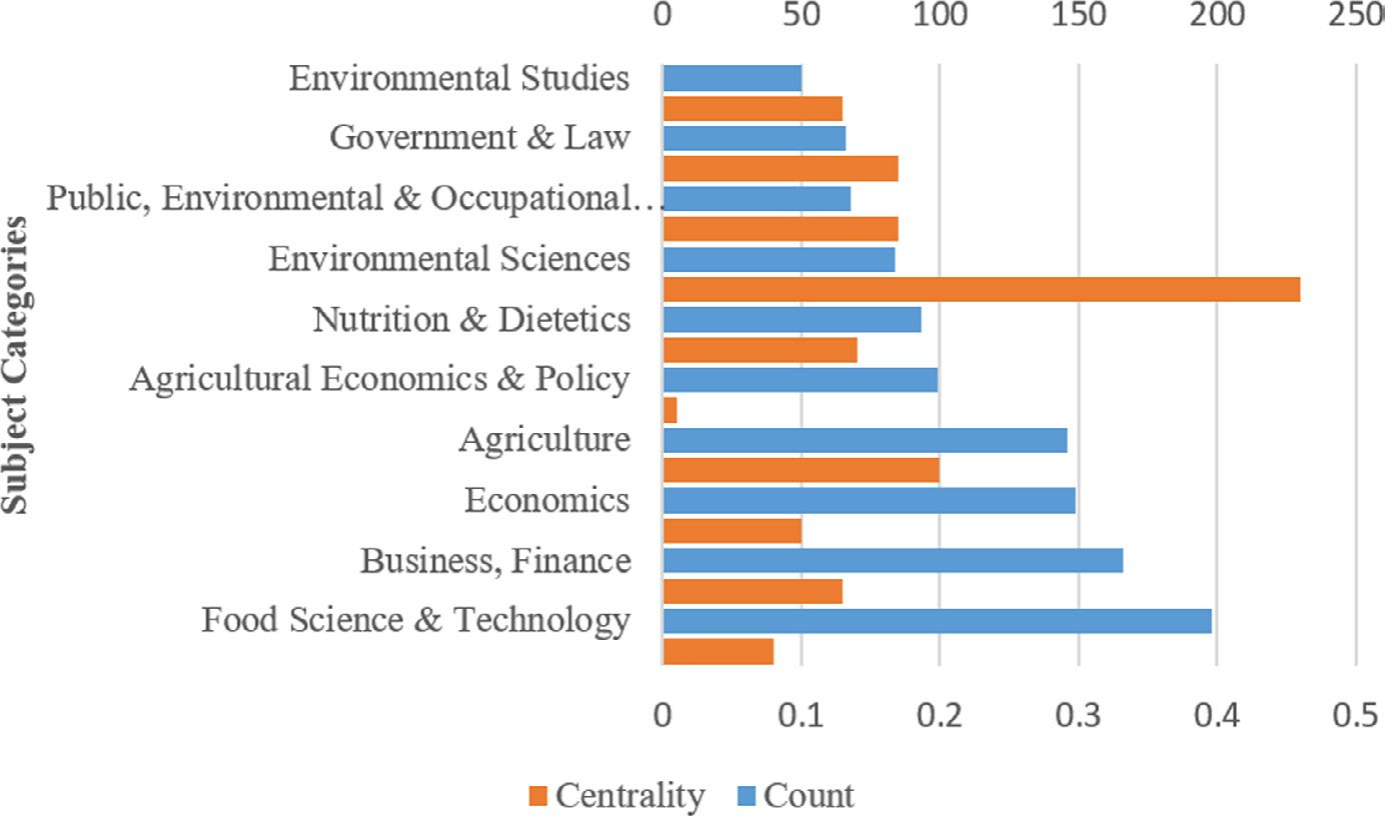
Count and centrality statistics for top 10 subjects

The discipline category analysis of the literature in this field is shown in Figure 4. The subject category information was extracted from tags of the WoS database by CiteSpace and then analyzed. Among all data sources, the top five subject categories with most publications were Food Science & Technology (198 articles), Business, Finance (166 articles), Economics (149 articles), Agriculture (146 articles), and Agricultural Economics & Policy (99 articles). In terms of the centrality index, the top five subject categories in literature pertaining to food safety governance were Environmental Sciences (0.46), Agriculture (0.20), Public, Environmental & Occupational Health (0.17), Government & Law (0.17), and Nutrition & Dietetics (0.14), indicating that food safety governance was closely related to environment, agriculture, public health, policies & regulations, and nutrition. It was concluded that food safety governance research had obvious characteristics of interdisciplinary integration.

### 3.2 Journal co‐citation and author co‐citation analyses

**FIGURE 5:**
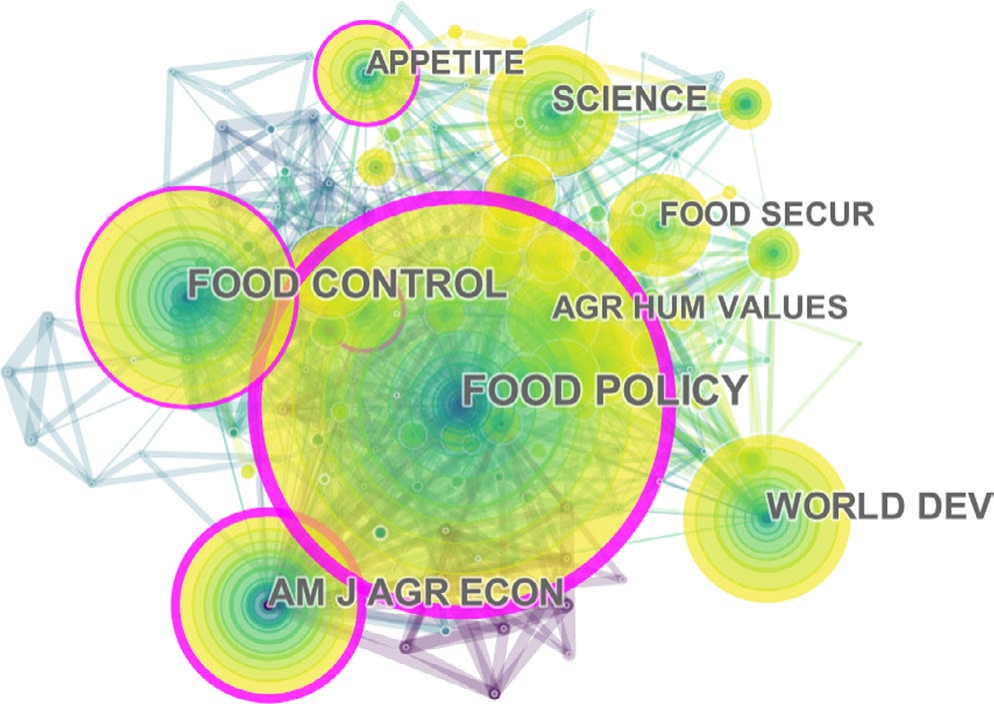
The network of scholarly journals in food safety governance from 1999 to 2019

The criteria of “core journals” were determined by the number of published articles and the frequency of co‐citations. According to the number of published articles, it was found that the top eight journals had an average impact factor (IF) of 2.63. Among them, the number of articles published by *Food Policy*, *Food Control,* and *Food Security* was much higher than that of other journals (Table 1). As another important indicator of the importance of journals, co‐citation frequency was positively correlated with journal quality. As seen from Figure 5, the top five co‐cited journals were *Food Policy* (318), *Food Control* (178), *American Journal of Agricultural Economics* (164), *World Development* (143), and *Science* (115). Through further exploration, it was found that the two journals, *Food Policy* and *Food Control,* had not only the largest number of articles published, but also a high co‐citation frequency. *Food Policy* ranked first in both indicators, representing its important position in relevant studies in the field of food safety governance. Although the two journals, *Sustainability* and *Int. J. Env. Res. Pub. Health* ranked in the forefront in terms of the number of published food safety governance‐related documents, due to the small number of total publications, they were not considered as the best choice for researchers to find the food safety governance‐related literature.

**TABLE 1:** The top eight scholarly journals

Rank	Publications	Journal	IF(Q) (2019)	Percentage	Journal total publication
1	50	*Food Policy*	4.189 (1)	6.76%	1,433
2	33	*Food Control*	4.258 (1)	4.46%	6,270
3	33	*Food Security*	2.095 (3)	4.46%	666
4	17	*Brit. Food J*.	2.102 (2)	2.30%	1778
5	14	*Agr. Hum. Values*	2.442 (1)	1.89%	678
6	12	*Sustainability*	2.576 (2)	1.62%	17,283
7	11	*Int. J. Env. Res. Pub. Health*	2.468 (3)	1.49%	13,463
8	11	*Food Drug Law J*.	0.905 (4)	1.49%	571

**FIGURE 6:**
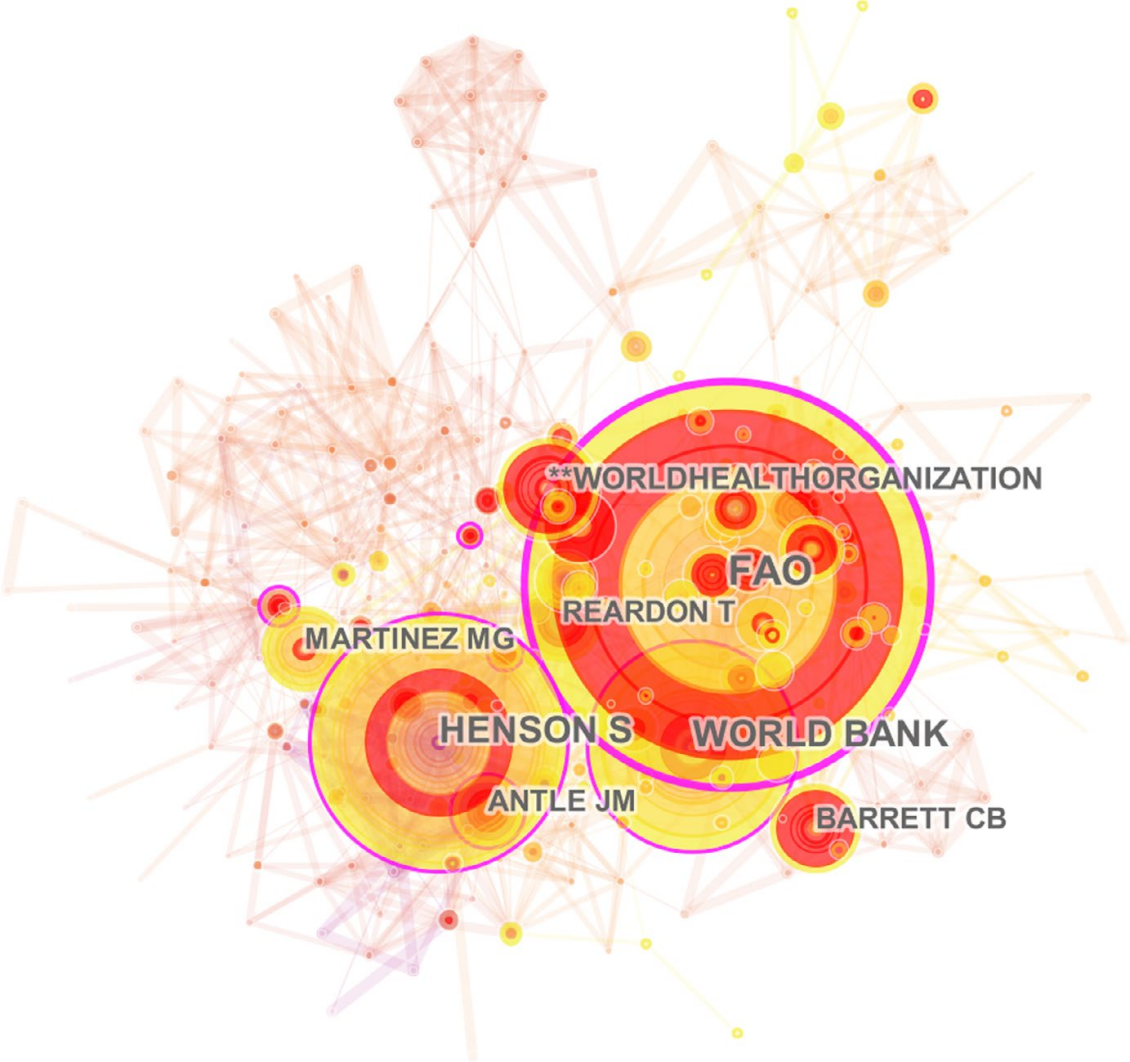
The network of the authors in food safety governance from 1999 to 2019

Figure 6 displays authors with highest co‐citation frequencies in this field. Among the top 10 authors, the co‐citation frequency of Food and Agriculture Organization of the United Nations (FAO), Henson S and World Bank was much higher than that of other authors, which showed that these authors had significant authority and high status in the research field of food safety governance. Author co‐citation burst indicated that many research articles by important authors were cited within a certain period. In this study, the burst values of Antle JM (1999–2009), Henson S (2014–2015), and Fulponi L (2014–2015) were found to be the highest (Table 2).

**TABLE 2:** The top 10 co‐cited authors

Rank	Co‐cited authors	Count	Co‐cited authors	Burst
1	FAO	134	Antle JM	10.32
2	Henson S	85	Henson S	7.52
3	World Bank	71	Fulponi L	7.34
4	WHO	35	FAO	6.67
5	Martinez MG	31	McMichael P	6.55
6	Barrett CB	31	WHO	6.48
7	Reardon T	30	USDA	6.19
8	Antle JM	27	Buzby JC	5.91
9	European Commission	21	Coleman‐Jensen A	5.58
10	Godfray HCJ	21	De Schutter O	5.57

### 3.3 Key node literature analysis

Key node literature was considered as those articles warranting most attention, as determined by the frequency of co‐citation. Through the analysis of 740 articles, 12 articles with the highest co‐citation frequency were obtained (Table 3). The following were the findings after the analysis of key node literature.

**TABLE 3:** Information of key node literature

Rank	Author (1st)	Year	Reference	Co‐citation	Centrality
1	Godfray	2010	Food security: The challenge of feeding 9 billion people	22	0.05
2	Martinez	2007	Co‐regulation as a possible model for food safety governance: Opportunities for public–private partnerships	16	0.13
3	Candel	2014	Food security governance: A systematic literature review	15	0.14
4	Barrett	2010	Measuring food insecurity	13	0.02
5	Rouviere	2012	From punishment to prevention: A French case study of the introduction of co‐regulation in enforcing food safety	12	0.08
6	Pei	2011	The China melamine milk scandal and its implications for food safety regulation	11	0.05
7	Mensah	2011	Implementation of food safety management systems in the UK	10	0.04
8	Ericksen	2008	Conceptualizing food systems for global environmental change research	10	0.03
9	Fulponi	2006	Private voluntary standards in the food system: The perspective of major food retailers in OECD countries	9	0.08
10	Ortega	2011	Modeling heterogeneity in consumer preferences for select food safety attributes in China	9	0.01
11	Liu	2013	Consumers' attitudes and behavior toward safe food in China: A review	9	0.03
12	Jia	2013	The national food safety control system of China—A systematic review	9	0.08

Firstly, food safety should be considered under a holistic umbrella: the access to adequate food at the demand side was influenced by social factors, such as family income, food prices, and unemployment rate (Barrett, 2010). Besides, to ensure the sustainable and effective supply of safe food, environmental issues such as carbon emission, land desertification, water pollution in the process of planting, breeding, processing, and production should be considered (Godfray et al., 2010). At present, the food system was not only a “farm‐to‐fork” process management, but also a complete system framework composed of food safety, environmental safety, and social welfare, which involved various environmental, social, political, and economic factors (Ericksen, 2008).

Secondly, the food safety management model had undergone the transformation from the separate formulation of the standards for public and private sectors to the joint supervision of public and private sectors. In the early stage, food retailers usually formulated and implemented private quality and moral standards stricter than government standards to ensure high‐quality reputation and customer loyalty (Fulponi, 2006). Martinez *et al*. pointed out the limitations of standards formulated by government and private sectors, proposed the model of joint management between public and private sectors, and theoretically identified the effectiveness of this model in food safety governance (Garcia Martinez et al., 2007). In addition, several subsequent studies had further verified that this governance model could benefit the private sector in practice. For example, it was found that the joint management model could increase the willingness of small‐ and medium‐sized food retailers to comply with laws and regulations. These studies can not only help them gain good reputation in domestic and foreign markets (Mensah & Julien, 2011), but can motivate food operators in the market (Rouviere & Caswell, 2012); however, it is worth noting that the current academic empirical test on co‐governance of food safety is insufficient (Candel, 2014).

Thirdly, the issue of Chinese food safety has received widespread attention, which was probably related to the large number of relevant research institutions and frequent food safety incidents in China. Some studies pointed out the deficiencies of food safety governance in China. For example, there was a large gap between China and European and American countries in law making, organization setting, supervision, implementation, technical support, and information exchange in food safety governance system (Jia & Jukes, 2013). Moreover, there was a need to eliminate the dependence of food safety on final product testing and strengthen the process management of standard setting, on‐site inspection, and food traceability (Pei et al., 2011). Meanwhile, the regularity of Chinese consumers' food safety preference needed to be considered. It was indicated that Chinese consumers had higher trust in the quality of food certified by the government compared with that without government certification (Ortega et al., 2011). In addition, although consumers had a strong willingness to buy safe food, they lacked the ability to identify safe food (Liu et al., 2013).

### 3.4 Popular research topics

**FIGURE 7:**
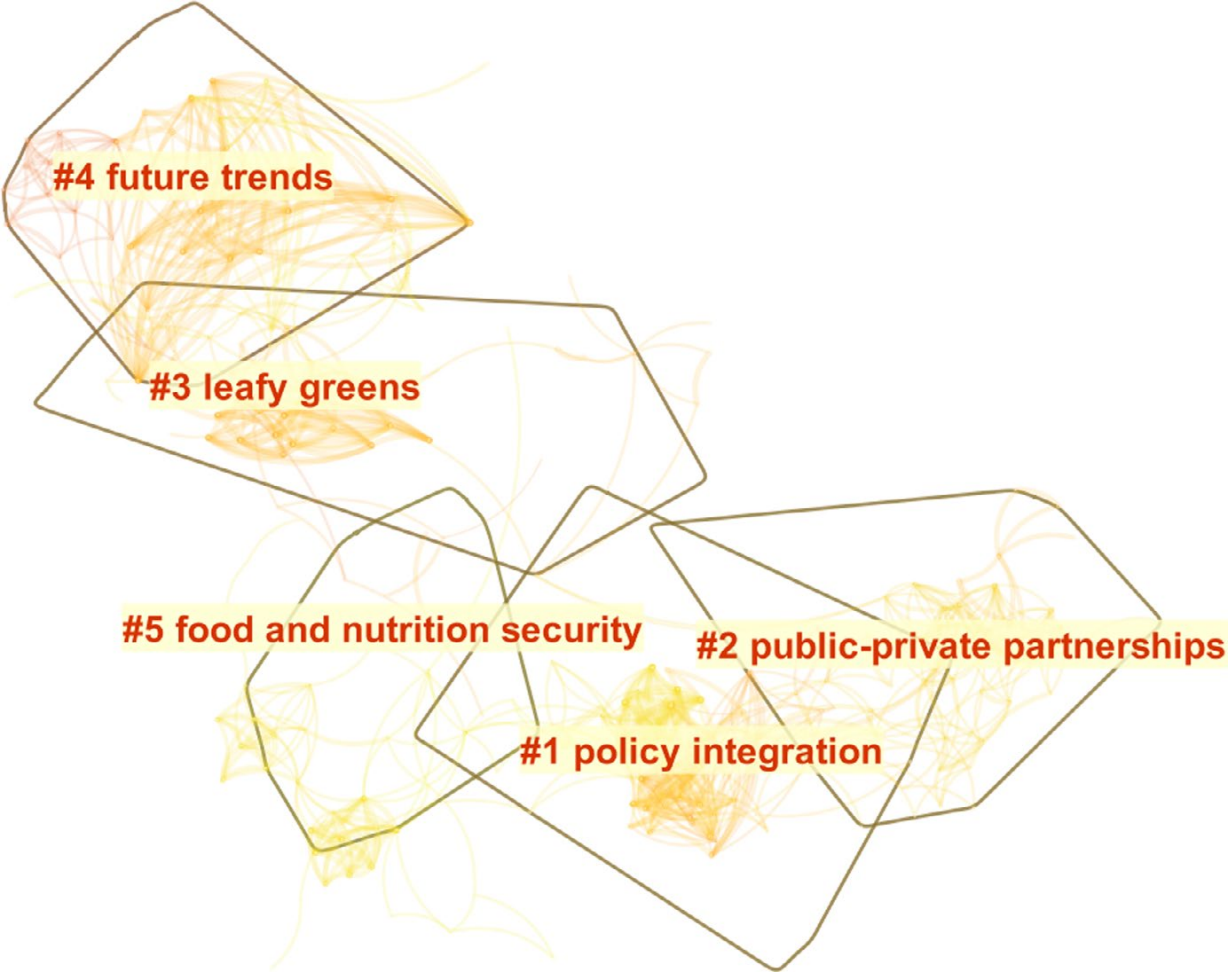
Clusters of knowledge domain in food safety governance from 1999 to 2019

Through cluster analysis, the research hot spots of food safety management were summarized. Node type was selected as Cited Reference. The Top N was selected as Top 50, which reflected the 50% term or document appeared most frequently each year. At the same time, set year per slice to 1. Set the threshold to (2, 2, 20) (4, 3, 20) (4, 3, 20). The names of the five largest clusters were extracted, namely Policy Integration, Public–private Partnerships, Leafy Greens, Future Trend, and Food & Nutrition Security. The results were presented in Figure 7. As shown in Figure 7, *N* = 615, *E* = 2,108, Density = 0.0112, Modularity *Q* = 0.852 (>0.3); Mean Silhouette = 0.4719 (>0.4), which indicated that the goodness of fit of the graph was good. The Modularity *Q* reflected whether the graph could be divided into clusters, and the Mean Silhouette reflected whether the relationship among the documents within each cluster was close enough (Chen et al., 2010).

#### 3.4.1 Cluster #1

The name of Cluster #1 was Policy Integration. The definition of the cluster name came from the log‐likelihood ratio (LLR) algorithm of CiteSpace. In Cluster #1, Mean Silhouette = 0.856 (>0.4), reflected the close relationship between the documents in the cluster. Policy Integration was defined as the cluster name because it ranked the first among all top terms (LLR = 8.72, *p*‐level = 0.005). The indicator and main literature information of Cluster #1 are shown in Table 4 below.

**TABLE 4:** Indicator and main literature information of Cluster #1

Cluster ID	Size	Silhouette	Top Terms (log‐likelihood ratio, *p*‐level)	Main Literature (1st Author)	Research methodology
1	52	0.856	Policy integration (8.72, 0.005)	Godfray (2010)	Literature analysis
				Barrett (2010)	Literature analysis
				Candel (2014)	Literature analysis
				Fernando (2015)	Empirical analysis
				Bloom (2015)	Empirical analysis
				Khalid (2016)	Empirical analysis
				Candel (2018)	Empirical analysis
				Pouliot (2018)	Literature analysis
				Mercure (2019)	Empirical analysis
				Venghaus (2019)	Empirical analysis

In this cluster, researches of food policy integration could be divided into two different categories. Firstly, some researchers concentrated on the policy integration within the food system. In the early stage, the formulation of food safety policies and standards mainly focused on a single link in the industrial chain; however, as the risks accumulated in the upstream reach of the food supply chain tend to break out in the downstream end, only focused on the food safety in a certain link in the supply chain cannot ensure the supply of safe food. Therefore, more concern had been received to the overall food supply chain from farm to fork to seek an optimal food policy. Research of this cluster has discovered the good experience of developed countries in food safety policy integration and some problems that have arisen in the process of food safety policy integration in developing countries. For example, Candel and Biesbroek (2018) analyzed the successful experience of EU food safety policy integration from four aspects: the policy frame, subsystem involvement, policy goals, and policy instruments (Candel & Biesbroek, 2018). Pouliot and Wang (2018) believed that the successful experience of food safety in the United States is a combination of government intervention and government incentives (Pouliot & Wang, 2018). However, some developing countries might have some problems in the process of food safety policy integration. For example, Fernando et al. (2015) found that government incentives did not seem to positively impact Malaysia's food safety governance system (Fernando et al., 2015). Bloom (2015) believed that the dispersion of Honduras food safety policy standards led to confusion between food safety and sustainability (Bloom, 2015). Secondly, some other researchers also focused on the relationship between the food system and other systems under policy integration. For example, Mercure et al. (2019) built a food‐energy‐water system under Brazil's policy integration (Mercure et al., 2019). Venghaus et al. (2019) broadened this system to a larger food‐energy‐water‐agricultural system to further explore the effects of policy integration on the EU food industry (Venghaus et al., 2019). This reflected the fact that the realization of food policy integration required improvement of coordination among different government authorities (Khalid, 2016).

#### 3.4.2 Cluster #2

The name of Cluster #2 was Public–private Partnerships. According to the LLR algorithm of CiteSpace, the Mean Silhouette in Cluster #2 was 0.887 (>0.4), reflected the close relationship between the documents in the cluster. Public–private Partnerships was defined as the cluster name because it ranked the first among all top terms (LLR = 9.03, *p*‐level = 0.005). The indicator and main literature information of Cluster #2 are shown in Table 5 below.

**TABLE 5:** Indicator and main literature information of Cluster #2

Cluster ID	Size	Silhouette	Top Terms (log‐likelihood ratio, *p*‐level)	Main Literature (1st Author)	Research methodology
2	49	0.887	Public–private partnerships (9.03, 0.005)	Ortega (2011)	Empirical analysis
				Rouviere (2012)	Empirical analysis
				Jia (2013)	Literature analysis
				Liu (2013)	Literature analysis
				Chen (2018)	Mathematical modeling
				Zhang (2014)	Mathematical modeling
				Holtkamp (2014)	Empirical analysis
				Veeck (2015)	Empirical analysis
				Yasuda (2015)	Empirical analysis
				Liu (2016)	Empirical analysis
				Duncan (2018)	Mathematical modeling

In this cluster, the participation of different governance entities and the interests between different governance subjects were analyzed in detail. Normally, the government was considered the most important governing sector in the food market. However, Candel (2014) argued that the main critique of the global governance of food security was that there was no truly authoritative and encompassing body with a mandate to address food security concerns across sectors (Candel, 2014). In addition to the government authority, other stakeholders such as consumer, news media, industrial association, third‐party certification, and consumer associations could also play increasingly prominent roles in food safety governance, which attracted extensive attention among researchers. For example, Duncan and Claeys (2018) believed that multi‐party governance bodies' participation could make food safety governance apolitical and enabled other stakeholders' roles to be realized (Duncan & Claeys, 2018). Zhang et al. (2014) believed that food safety governance under third‐party supervision included five types: media exposure, third‐party certification, consumer association supervision, social movements promoted by nongovernmental organizations, and industry association supervision, among which the media were the most critical sector (Zhang et al., 2014). Indeed, in recent years, the media's role has become more prominent in food safety governance. For example, Holtkamp et al. (2014) believed that when some official data on food safety governance were missing, the analysis of food safety issues under media reports would be an effective supplement (Holtkamp et al., 2014). Liu and Ma (2016) found that the media's food scandal was not significantly related to the public's level of concern about food safety risks. To this end, more attention should be emphasized on public's awareness of food safety, and the media's magnification of public food safety concerns should be eased (Liu & Ma, 2016). Of course, the participation of food safety co‐governance was inseparable from citizens. Veeck et al. (2015) believed that citizens' participation and understanding of citizens' food safety risks are more helpful for citizens to actively contribute to food safety governance issues and promote governance institutions' policy promotion (Veeck et al., 2015). However, the social co‐governance of food safety was not always effective. For example, Yasuda (2015) found that the massive production system, unwieldy bureaucracy, and geographic size in China posed regulators with a more fundamental policy challenge (Yasuda, 2015).

#### 3.4.3 Cluster #3

The name of Cluster #3 was Leafy Greens. According to the LLR algorithm of CiteSpace, the Mean Silhouette in Cluster #3 was 0.961 (>0.4), reflected the close relationship between the documents in the cluster. Leafy Greens was defined as the cluster name because it ranked the first among all top terms (LLR = 5.80, *p*‐level = 0.05). The indicator and main literature information of Cluster #3 are shown in Table 6 below.

**TABLE 6:** Indicator and main literature information of Cluster #3

Cluster ID	Size	Silhouette	Top Terms (log‐likelihood ratio, *p*‐level)	Main Literature (1st Author)	Research methodology
3	41	0.961	Leafy greens (5.80, 0.05)	Ivey (2012)	Empirical analysis
				Naziri (2014)	Empirical analysis
				Zhou (2015)	Empirical analysis
				Kirezieva (2015)	Empirical analysis
				Kirezieva (2016)	Empirical analysis
				Choi (2016)	Empirical analysis
				Hernandez (2018)	Empirical analysis
				Narrod (2009)	Empirical analysis

Through the analysis of top terms, it was found that supply chain security and quality of vegetables and fruits were the most concern by scientific research. With popular attention to green and healthy diets in recent years, fruit and vegetables have gradually become popular; however, the whole supply chain of fruit and vegetable, from planting and processing to sales, was vulnerable to contamination by pesticides and microorganisms. Compared with meat, agricultural products such as fruits and vegetables were usually eaten directly by consumers without cooking; thus, food‐borne diseases and emergencies associated with them occurred frequently. Most of the researches were carried out from the perspective of exploring the impact of vegetable and fruit supply chain security. Hernandez‐Rubio et al. (2018) believed that whether large retailers could establish strict wholesale quality control was a decisive factor in the food safety level of the supply chain of French and Spanish fruit and vegetable wholesalers (Hernandez‐Rubio et al., 2018). Kirezieva et al. (2016) studied the impact of strawberry supply market security in Belgium and the Netherlands from hierarchy‐like governance (Kirezieva et al., 2016). Besides, researchers also explored the relationship between the European vegetable supply chain's security with agriculture, climate, markets, and public policies (Kirezieva et al., 2015). Naziri et al. (2014) believed that technical assistance from professionals, the education level of employees, and the implementation of public training programs could have an important impact on the safety of Vietnamese vegetable production (Naziri et al., 2014). A study of American restaurant kitchen employees handling green leafy vegetable food safety showed that employees' food safety behaviors vary based on factors such as operation size, cuisine, and operation type (Choi et al., 2016). Zhou et al. (2015) compared three different production models of farmer cooperatives, agricultural companies, and family farms in a specific area of southern China and found that the scale of production positively affects the quality and safety control of fruits and vegetables (Zhou et al., 2015). The evaluation of quality and safety of fruit and vegetable food supply chain could provide practical guidance for food practitioners' standard behaviors.

#### 3.4.4 Cluster #4

The name of Cluster #4 was Future Trend. According to the LLR algorithm of CiteSpace, the Mean Silhouette in Cluster #4 was 0.942 (>0.4), reflected the close relationship between the documents in the cluster. Future Trend was defined as the cluster name because it ranked the first among all top terms (LLR = 7.46, *p*‐level = 0.01). The indicator and main literature information of Cluster #4 are shown in Table 7 below.

**TABLE 7:** Indicator and main literature information of Cluster #4

Cluster ID	Size	Silhouette	Top Terms (log‐likelihood ratio, *p*‐level)	Main Literature (1st Author)	Research methodology
4	39	0.942	Future trend (7.46, 0.01)	Tscharntke (2012)	Literature analysis
				Nesbitt (2014)	Empirical analysis
				Young (2015)	Literature analysis
				Sivaramalingam (2015)	Literature analysis
				Burke (2016)	Empirical analysis
				King (2017)	Literature analysis
				Wu (2018)	Literature analysis
				Mylona (2018)	Literature analysis
				Nayak (2019)	Literature analysis

Researchers have different understandings of the future trend in the research field, but their common goal is to improve the level of food safety governance. For example, based on the findings of *the EU Food Safety and Nutrition Outlook in 2050*, Mylona et al. (2018) discussed the concerns of future food policies. The researcher believed that increasing food production and reducing food waste were considered as two main efforts to achieve food security in the future (Mylona et al., 2018). King et al. (2017) believed that future climate change, population growth, population aging, and increased food demands were also considered as challenges in future research (King et al., 2017). Hence, it was believed that future research on global food safety would be viewed from a more systematic perspective and would adopt more complex methods because this would help further understand the interconnectedness of food systems and reduce food security risks (Nayak & Waterson, 2019; Wu et al., 2018b). Apart from the systematic research on food safety governance, strengthened food safety education would also become an essential means of food safety governance. The Internet and social media expanded information transmission and provided a prerequisite for food safety governance (Nesbitt et al., 2014). Young et al. (2015) believed that studies of the intervention and effectiveness of food safety education in developed countries were currently at the forefront (Young et al., 2015). Since effective education strategies were critical to change consumers' food safety attitudes and behaviors, future research would focus on consumers' food safety attitudes and behaviors (Burke et al., 2016; Sivaramalingam et al., 2015). To this end, it was possible to increase the popular attention to food safety knowledge through public education and training.

#### 3.4.5 Cluster #5

The name of Cluster #5 was Food and Nutrition Security. According to the LLR algorithm of CiteSpace, the Mean Silhouette in Cluster #5 was 0.942 (>0.4), reflected the close relationship between the documents in the cluster. Food and Nutrition Security was defined as the cluster name because it ranked the first among all top terms (LLR = 13.15, *p*‐level = 0.001). The indicator and main literature information of Cluster #5 are shown in Table 8 below.

**TABLE 8:** Indicator and main literature information of Cluster #5

Cluster ID	Size	Silhouette	Top Terms (log‐likelihood ratio, *p*‐level)	Main Literature (1st Author)	Research methodology
5	38	0.942	Food and nutrition security (13.15, 0.001)	Caswell (1999)	Literature analysis
				Qureshi (2015)	Empirical analysis
				Handford (2016)	Literature analysis
				Adhikari (2017)	Empirical analysis
				Mylona (2018)	Literature analysis
				Sparling (2019)	Empirical analysis
				Walls (2019)	Literature analysis

Food safety must accompany food and nutrition security (Chan, 2014). After food quantity safety is guaranteed, some studies in recent years have begun to turn their perspectives on the nutritional safety of food. Assuring the quality of food products, especially their safety and nutrition levels, is an increasing focus for governments, companies, and international trade bodies (Caswell, 1998). Existing researches on food nutrition safety were mostly related to public health. In cluster 5, researchers explored the relationship between food nutrition security with physical and mental health. Sparling et al. (2019) found that food nutrition safety was associated with the probability of depression. Via the consumption of dairy, eggs, fish, vitamin A‐rich, and vitamin C‐rich foods was associated with reduced depression rates (Sparling et al., 2019). Adhikari et al. (2017) believed that factors such as the deterioration of the local food system, changes in eating habits, and lack of understanding of the use and nutritional value would affect the nutrition safety and health of residents (Adhikari et al., 2017). Another research conducted by Handford et al. (2016) aimed to investigate the impact of milk fraud on nutrition and food safety and pointed out the potential adverse effects of consuming adulterated milk on human health (Handford et al., 2016).

In addition to analyzing the contents of primary documents in the five top clusters, we also found that researchers who focused on food safety governance were more likely to use research methods such as empirical analysis, literature analysis, and mathematical modeling analysis. Among these methodologies, empirical analysis was used to determine critical factors that would affect food safety governance from different governance stakeholders. Methods such as case studies, econometric analysis, and structural equation modeling were used most (Kirezieva et al., 2016; Ortega et al., 2011; Rouviere & Caswell, 2012). The research used literature analysis as the method mainly based on the perspective of the literature review, such as discussing a specific issue of concern in regional food safety governance or constructing a theoretical framework for food safety governance (Barrett, 2010; Candel, 2014; Godfray et al., 2010). The literature used mathematical modeling as the method mostly cared about food safety governance participation of different subjects. It used game theory to construct the cooperation and game relationship between different governance subjects under the food safety governance environment and between the governance subjects and the governed entities (Chen et al., 2018; Duncan & Claeys, 2018; Zhang et al., 2014).

### 3.5 Research trends

**FIGURE 8:**
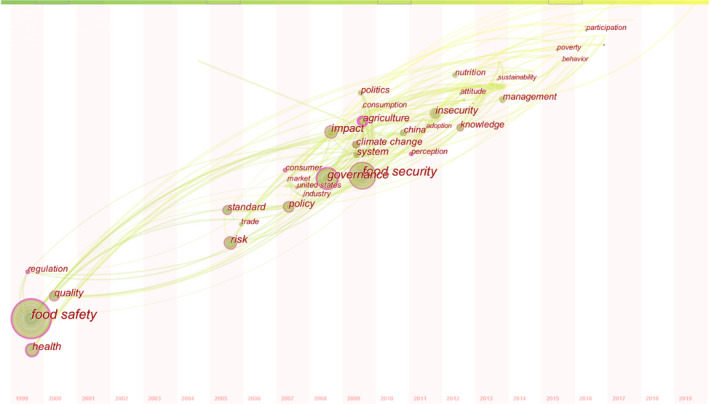
Keyword collinear time zone diagram in food safety governance from 1999 to 2019

The evolution and trend of the research were analyzed based on the collinear time zone diagram of keywords and the keywords' burst value. The node type in CiteSpace was selected as a keyword, TopN was set to 20, and the time slice was 1. The collinear time zone diagram of keywords was drawn in Figure 8. According to research characteristics, food safety management can be divided into the following three stages.

Stage 1 (1999–2007): formulation of food safety governance policies, regulations, and standard systems. According to the collinear time zone diagram of keywords, the high‐frequency words in the first stage included food safety, quality, health, regulation, standard, and policy. Since the construction of the food safety governance system in developing countries was in its infancy at this stage, research into food safety governance was focused on developed economies such as European countries. At this stage, the optimization and improvement of existing food safety regulations and standards had become a common concern. For instance, severe beef and pork quality issues and food safety risks in all supply links were highly concentrated (Rajic et al., 2007; Sargeant et al., 2007). Furthermore, the construction of food control system and food supervision institutions (Halkier & Holm, 2006; Neeliah & Goburdhun, 2007), the formulation of food testing standards (Starbird, 2005), and the optimization of quality assurance mechanism (Hobbs et al., 2002) were also used as useful tools for food safety enhancement.

Stage 2 (2007–2013): the establishment of system engineering model of food safety governance. According to the collinear time zone diagram of keywords, the main high‐frequency words in the second stage included consumer, market, climate change, industry, and system. Different from Stage 1, research in Stage 2 began to gradually connect the relationship of different stakeholders in the food supply chain. Research at this stage was more concentrated on the whole process of food safety management from farmland to table to study food safety governance issues (Ericksen, 2008) and further explored the food system from a multi‐dimensional perspective (Barrett, 2010; Godfray et al., 2010). Besides, it was also found that food safety governance officers should pay heed to the cooperative, win–win model with multi‐subject participation in food safety governance (Narrod et al., 2009; Trienekens & Zuurbier, 2008).

Stage 3 (2013–2019): the formation of food safety stakeholders and sustainable development concept. According to the collinear time zone diagram of keywords, the high‐frequency words in the third stage included participation, behavior, knowledge, and sustainability. The research in this period focused on the multi‐subject participation in food safety governance, consumer behavior, food safety education, and sustainable development of food safety (Chen et al., 2018; Ma & Liu, 2019; Nesbitt et al., 2014; Young et al., 2015). Investigation of consumer behavior and their level of education with regard to food safety can help government departments to better understand consumer demand for safe food. This would help policymakers improve food policies aligned better with market laws and would also be more conducive to the sustainable development of food safety governance (Lim et al., 2016).

**FIGURE 9:**
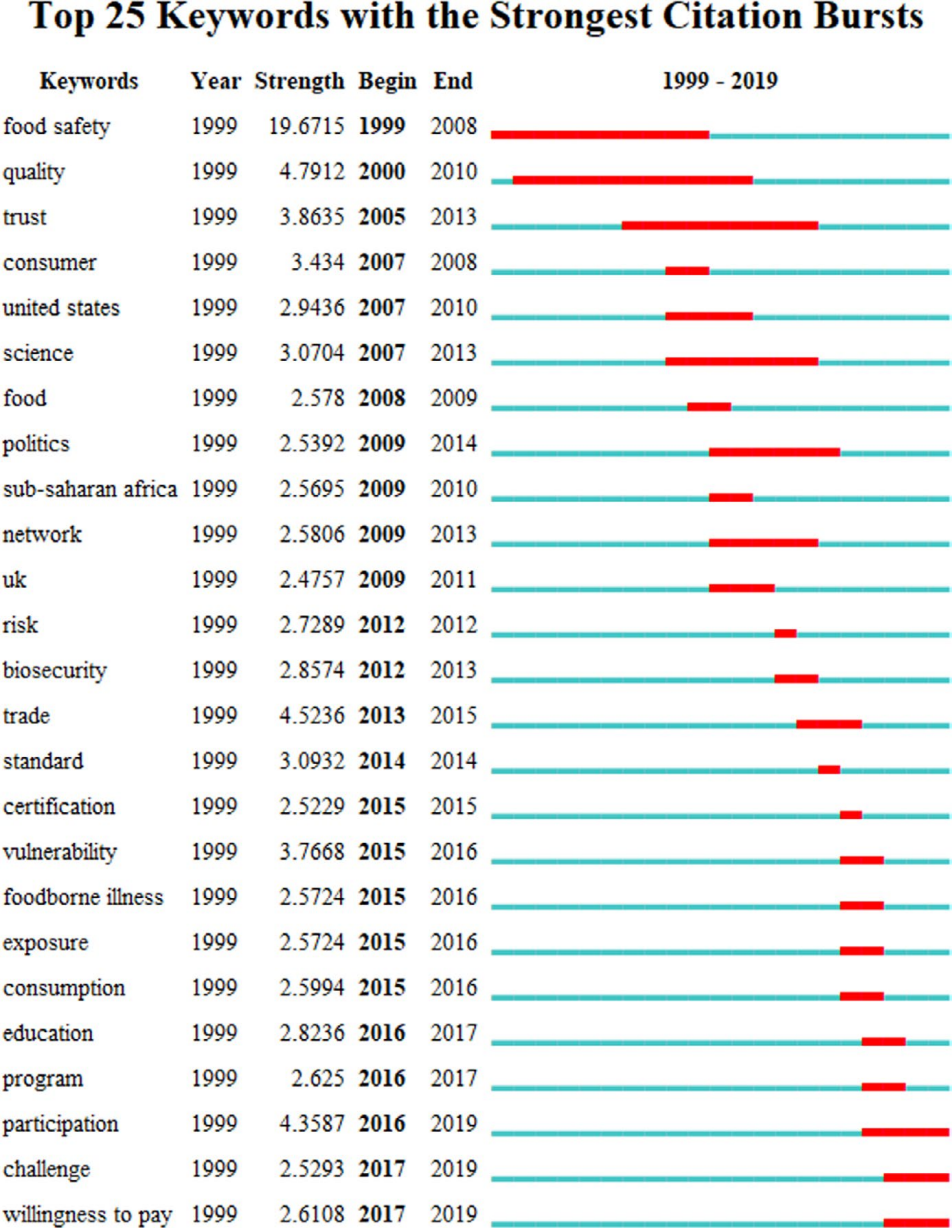
Top 25 keywords with the strongest citation bursts in food safety governance

Then, the top 25 keywords with the strongest citation bursts were found: among these keywords, “participation”, “willingness to pay”, and “challenge” were important keywords which showed high‐intensity citation bursts in 2019. These three keywords were further used to analyze the current research trends in food safety governance (Figure 9).

Firstly, “participation” was an important burst keyword from 2016 to 2019 with a strength of 4.36. The current research focused on the participation of food safety management. After the co‐governance model was proposed, more stakeholders in the food market began to participate in food safety governance. In addition to government, consumers, industry associations, consumer associations, and news media were gradually becoming important participants in food safety governance (Duncan & Claeys, 2018; Zanella et al., 2018; Zhu et al., 2019).

Secondly, “willingness to pay” was another burst keyword from 2017 to 2019 with a strength of 2.61, indicating that consumer willingness to pay for safe food is a research hot spot and frontier at this stage. The study of consumer willingness to pay not only helped to understand consumer awareness and concern about food safety, but also laid a foundation for the optimization and adjustment of food safety policies. There were various factors influencing consumer willingness to pay for safe food, such as the occurrence of food safety incidents and the lack of food safety education (Li et al., 2018). Based on this, it was necessary to improve consumer awareness and knowledge of food quality and safety certification, especially in developing countries (My et al., 2017). To this end, government should also strengthen food safety education and training to guide consumers to buy safe, high‐quality food (Bloom, 2015).

The third burst word was “challenge” from 2017 to 2019, with a strength of 2.53. It focused on the challenges of current food safety issues, such as population explosion, inadequate supply of safe food (Fouilleux et al., 2017; Qureshi et al., 2015), coordination of the correlation between food safety and ecological environment system, and balancing the relationship between food safety, biodiversity, water security, and energy security (Glamann et al., 2017; Scott et al., 2018) on the premise of ensuring food safety. Besides, future food safety governance challenges would also reflect the food safety governance issues in internet technology and public health pandemics. For instance, the deep integration of internet technology and the food industry enabled increasing number of consumers to shift their food purchasing habits offline to online. Compared with buying food in physical stores, online food transactions increased supply chains (online purchases and offline distribution), information asymmetry, risk occurrence probability, and governance bodies (introduction of third‐party platforms), which caused more severe food safety issues (Shen & Wei, 2020). Therefore, online food safety governance would become a new trend in food safety governance research (Yu et al., 2020; Zhao et al., 2017). On the other hand, food safety issues under public health pandemics received widespread attention. For example, the Food and Agriculture Organization of the United Nations warned that under the COVID‐19 epidemic, the world was facing the worst food crisis in 50 years, and 700 million people worldwide were in starvation. Therefore, the analysis of the impact of sudden public health events on food safety governance would also become the direction of future research. The food supply chain disruption, food and water safety, food monitoring technology, malnutrition, and consumer food behavior would become new challenges under the COVID‐19 pandemic (Knorr & Khoo, 2020). Online booking, centralized procurement, and community distribution would become practical to reduce cross‐infection risk under the pandemics to meet urban residents' food safety needs (Guo et al., 2020).

### 3.6 Regional analysis based on income level

Through the combination of current literature, it was found that many scholars had explored food safety governance issues based on their familiar regions. Hence, this section would focus on food safety governance issues in different regions based on economic income levels. According to the World Bank's 2019 guidelines, a country whose per capita Gross National Income (GNI) less than 1,035 USD was considered a lower‐income country. GNI over 12,535 USD belonged to a higher‐income country. GNI between 1,035 and 12,535 USD belonged to a middle‐income country (http://data.worldbank.org/data‐catalog/world‐development‐indicators). Based on these indicators, we chose sub‐Saharan Africa as lower‐income regions; China, India, Malaysia, and Vietnam as middle‐income countries; and the United States, the United Kingdom, Australia, and New Zealand as higher‐income countries.

Firstly, sub‐Saharan Africa was chosen as the representative of lower‐income economics. It was found that although some lower‐income countries in sub‐Saharan Africa had learned from the food safety governance systems of developed countries, their governance systems might not play a significant role in the implementation process. For example, the local government's fragmented institutional settings and fuzzy division of responsibilities at the Zimbabwe government's food safety governance level lacked an exact and coordinated mechanism (Pswarayi et al., 2014). The lack of a food safety governance system made lower‐income countries still troubled with food supply safety issues (James & Zikankuba, 2018). Although the export of fresh products provided opportunities for some coastal sub‐Saharan African countries' economic development, the lack of comprehensive food safety industry standards and transparent governance systems hindered food exports (Unnevehr, 2000). With increasing globalization, rapid urbanization, and rapid technological innovation, the food safety governance gap between sub‐Saharan Africa and other countries might further widen (Pinstrup‐Andersen, 2000). At present, most research into food safety governance in Africa linked food safety issues with social issues such as public health (Morse et al., 2018), environmental protection (Schlenker & Lobell, 2010), immigration, ethnic conflict, and pest control (Murage et al., 2015).

Then, the middle‐income countries in this research were taken as examples, including India, China, Malaysia, and Vietnam. Among these countries, India and China were larger economies with fast‐growing development, while Malaysia and Vietnam were small middle‐income economies. Through the literature of food safety governance research in these countries, it was possible to discover the current status and existing food safety governance problems in middle‐income countries. Although these countries had their own food safety governance systems, the food safety problem remained severe and the systems were still not perfect (Chen et al., 2014). For India, although the country promulgated the Food Safety and Standard Law in 2006, and implemented The Public Distribution System to reduce food safety risks, there remained low efficiency of food safety governance, and the environmental pollution problem under food safety governance was not effectively resolved (George & McKay, 2019). In India, food security and agricultural productivity were mainly affected by climate change, resource degradation, and monoculture of traditional agriculture (Ghosh et al., 2010). The relationship between food safety governance and arable lands, water resources, and planting strategy choices received more attention in current research (Bhanja & Mukherjee, 2019; Khanal & Mishra, 2017). China's food safety issues received increasing attention from the academic community in recent years, and discussions on food safety governance issues became more comprehensive. After the contaminated milk powder incident in 2008, the Chinese Government promulgated the Food Safety Law in 2009 to strengthen supervision of the dairy industry (Chung & Wong, 2013). In the following period, topics such as institutional systems, industry incentives, and safety standard formulation had attracted academic attention (Chen et al., 2015; Pei et al., 2011); however, affected by environmental pollution, excessive use of pesticides, backward technology, low‐risk awareness, lack of social responsibility, and the pursuit of short‐term economic benefits, China's food safety problems remained serious (Guo et al., 2019; Liu et al., 2018; Unnevehr & Hoffmann, 2015; Wen et al., 2017; Zhang et al., 2019). In addition, factors such as corporate governance structure (Zhou et al., 2015), food production scale (Parker et al., 2016), and different regional aspects (Liu & McGuire, 2014) would also affect the performance food safety supervision. After the promulgation of the new version of the Food Safety Law in 2015, China's food safety governance system emphasized on the transition from single government governance to a common social governance. In addition to government intervention, the joint supervision of multiple stakeholders including the news media, the public, and consumer associations became the focus of academic attention (Han & Yan, 2019; Yasuda, 2015; Zhu et al., 2019). Some other middle‐income economies, for example, Malaysia and Vietnam, also established their food safety governance system in 2009 and 2011. Due to the economic characteristics, food safety governance research in these two countries focused on the traditional farm produces fairs, traditional processing companies, and small catering companies (Samapundo et al., 2016). Standardized employee operation of food processing enterprise and catering service was also considered the focus of food safety governance in Malaysia and Vietnam (Abdul‐Mutalib et al., 2015; Sinclair et al., 2019; Wertheim‐Heck et al., 2015). Through the above analysis, it was not difficult to find that the implementation of the food safety governance system in the middle‐income countries mostly occurred in the past ten years. Although the food safety governance system improved to a certain extent compared with lower‐income countries, food safety governance in middle‐income economies still faced with severe foodborne diseases (Fernando et al., 2014).

Compared with lower‐income and middle‐income countries, higher‐income countries such as the United Kingdom, the United States, Australia, and New Zealand had some familiar characteristics in food safety governance. Firstly, higher‐income countries usually established mature food safety governance systems. For example, the United States passed food safety‐related bills as early as 1906. Great Britain also established a complete basic framework of food safety laws in 1990 (Luning et al., 2015). The Australian and New Zealand governments worked together through Food Standards Australia New Zealand (FSANZ) and other co‐operative agreements to improve food safety governance (Ghosh, 2014). Under these mature regulations, most food companies could comply with regulations and actively respond to food safety risks under a food safety management system (Mensah & Julien, 2011). Secondly, compared with lower‐income and middle‐income countries that were still concerned with food quantity safety, food safety governance in higher‐income countries mainly focused on food quality safety, food technology safety, and food nutrition safety. Current uncertainties in food safety governance in higher‐income countries were centralized on issues such as microbial contamination (Ivey et al., 2012), genetically modified technology (Castellari et al., 2018), and imported food safety (Keener et al., 2014): food safety governance research therein involved more discussion of food nutrition safety and diet health (Laska et al., 2012). Thirdly, compared with lower‐income and middle‐income countries, consumers in higher‐income countries were more involved in food safety governance. Higher‐income countries had relatively sound consumers' food safety governance participation. Consumers' voices on perceived accountability, transparency, traceability, and effectiveness of existing food governing structures were conducive to policymakers to consider food safety governance concerns from the consumer's perspective. It was also more conducive to improve the overall level of food safety governance (Devaney, 2016). Although some middle‐income countries also did some research on consumer food safety surveys, they mainly focused on consumers' preferences for food safety, and there was little research on the consumers' evaluation of food safety governance systems (Barling et al., 2009). Government departments in higher‐income countries also made great efforts in consumers' education on food safety, guided consumers to improve their ability of identify unsafe food, cultivated consumers' healthy food safety dietary concepts, and led consumers actively contribute to the government's supervision (Sivaramalingam et al., 2015; Young et al., 2015).

## 4 CONCLUSION

This study summarized researches concerning food safety governance collected by the Web of Science Core Collection. Through the analysis of research status, research trends, and research hot spots, several conclusions were drawn. As to the annual publishing trend, the number of articles about food safety governance had been increasing rapidly after 2008, with a surge in the number of articles in 2009 and 2015. As to published countries and academic institutes, The United States, The United Kingdom, and China had made significant contributions in this field. Wageningen University, International Food Policy Research Institute and University of Guelph were the three most influential institutions in the field, with the largest number of publications in food safety governance research; however, the degree of cooperation between academic institutions was not high. Research into food safety governance field involved cross‐disciplinary studies in Food Science & Technology, Business, Finance, Agriculture, Environmental Sciences, and other disciplines. *Food Policy*, *Food Control*, and *Food Security* were the top three journals with most publications. *Food Policy* and *Food Control* were the journals with the greatest impact in this cognate area. The FAO, Henson S, and World Bank had made significant contributions to the field.

The research hot spots mainly covered the food safety policy integration and the public–private partnership of food safety governance. As for research trends, the food safety co‐governance, the willingness to pay for safe food, and the challenges faced by food safety governance (including online food governance and food safety under pandemics) remained the focus of future research. The development of food safety governance theory could be divided into three processes: the separate formulation of the standards for public and private sectors, the joint implementation of the standards, and social co‐governance by multiple sectors. Under the global food safety governance system, scholars believed that the joint participation of government, consumer, media, and industry associations in food safety governance was required. When focused on food safety governance in countries with different income levels, it showed that lower‐income countries were concerned about food quantity and supply safety; middle‐income countries focused on the development of sound government systems. In contrast, higher‐income countries emphasized food safety nutrition governance and the governance participation from consumers.

## CONFLICT OF INTEREST

The authors declare no conflict of interest.

## ETHICAL APPROVAL

This study does not involve any human or animal testing. Written informed consent was obtained from all study participants.
